# Excess Circulating Angiopoietin-2 May Contribute to Pulmonary Vascular Leak in Sepsis in Humans

**DOI:** 10.1371/journal.pmed.0030046

**Published:** 2006-01-24

**Authors:** Samir M Parikh, Tadanori Mammoto, Aylit Schultz, Hai-Tao Yuan, David Christiani, S. Ananth Karumanchi, Vikas P Sukhatme

**Affiliations:** 1Department of Medicine, Beth Israel Deaconess Medical Center, Harvard Medical School, Boston, Massachusetts, United States of America; **2**Occupational Health Program, Harvard School of Public Health, Boston, Massachusetts, United States of America; National Heart and Lung InstituteUnited Kingdom

## Abstract

**Background:**

Acute respiratory distress syndrome (ARDS) is a devastating complication of numerous underlying conditions, most notably sepsis. Although pathologic vascular leak has been implicated in the pathogenesis of ARDS and sepsis-associated lung injury, the mechanisms promoting leak are incompletely understood. Angiopoietin-2 (Ang-2), a known antagonist of the endothelial Tie-2 receptor, was originally described as a naturally occurring disruptor of normal embryonic vascular development otherwise mediated by the Tie-2 agonist angiopoietin-1 (Ang-1). We hypothesized that Ang-2 contributes to endothelial barrier disruption in sepsis-associated lung injury, a condition involving the mature vasculature.

**Methods and Findings:**

We describe complementary human, murine, and in vitro investigations that implicate Ang-2 as a mediator of this process. We show that circulating Ang-2 is significantly elevated in humans with sepsis who have impaired oxygenation. We then show that serum from these patients disrupts endothelial architecture. This effect of sepsis serum from humans correlates with measured Ang-2, abates with clinical improvement, and is reversed by Ang-1. Next, we found that endothelial barrier disruption can be provoked by Ang-2 alone. This signal is transduced through myosin light chain phosphorylation. Last, we show that excess systemic Ang-2 provokes pulmonary leak and congestion in otherwise healthy adult mice.

**Conclusions:**

Our results identify a critical role for Ang-2 in disrupting normal pulmonary endothelial function.

## Abstract 2

A series of studies in sepsis patients, mice, and cultured endothelial cells suggest that excess levels of Ang-2 can provoke pulmonary leak and congestion and might be responsible for sepsis-associated Acute Respiratory Distress Syndrome.

## Introduction

Sepsis is characterized by a systemic inflammatory response to a microbial pathogen. In the United States, this illness accounts for 2%–3% of all hospital admissions (∼650,000 per year to major hospitals), carries a mortality of ∼30%, and has estimated annual direct costs in excess of $16 billion [1]. Although the mortality associated with sepsis has declined in the past 20 years [2], it remains a common and deadly condition. Among individuals with sepsis, those who develop shock and multi-organ dysfunction are at greatest risk of death [3].

Capillary permeability is a tightly regulated feature of microcirculation in all organ beds and is fundamentally altered in sepsis, resulting in net extravasation of fluid out of the vascular space and into tissues. A dramatic manifestation of this phenomenon is acute respiratory distress syndrome (ARDS), a complication that occurs in up to 40% of patients with sepsis and is marked by leakage of fluid out of pulmonary capillaries and into alveolar septa and air spaces [4]. Excess extravascular fluid in the lung impairs gas exchange across the alveolar membrane and decreases lung compliance. ARDS associated with sepsis has been correlated with adverse clinical outcomes, including ∼40% mortality [5–7].

Endothelial barrier integrity is thought to be a balance between (1) contractile forces within endothelial cells (ECs) that permit paracellular leakage by creating intercellular gaps and (2) adhesive forces between ECs that restrict such gaps. Myosin light chain (MLC), in its phosphorylated form (MLC-p), mediates contraction in a wide variety of cells, including the microvascular endothelium. An increase in contractile force within ECs—via MLC-p—is associated with increased permeability [8,9]; conversely, blockade of MLC-p reduces trans-endothelial permeability [10].

Angiopoietin-1 (Ang-1) and angiopoietin-2 (Ang-2) are peptide ligands that bind the Tie-2 receptor tyrosine kinase found primarily on ECs. They were first identified as an agonist/antagonist pair necessary for embryonic vascular development [11,12]. Ang-1 appears to promote vessel stability [12,13] by recruiting pericytes to nascent blood vessels [14] and preserving cell–cell contacts [15]. Ang-1 may also have an anti-inflammatory action by signaling the down-regulation of surface-adhesion molecules such as VCAM-1 and E-selectin [16]. Ang-1, expressed in supraphysiologic concentrations, appears to function as an anti-permeability agent in rodent dermal capillaries [17]. Disruptions in the Tie-2 signaling pathway can occur in developmental lung disorders [18], and expression of Tie-2 in quiescent adult tissues is highest in the lung [19,20]. Together these reports suggested (a) that an imbalance in Tie-2 signaling could arise in the inflammatory milieu of sepsis, and (b) that, should such an imbalance arise, the lung might be preferentially affected.

We therefore hypothesized that excess Ang-2 signaling may occur in sepsis, promoting pulmonary vascular leak through up-regulation of MLC-p. We explored this question in several settings: (1) humans with sepsis in whom circulating Ang-2 levels were measured, (2) EC monolayers in which signaling and structural responses to sepsis serum from humans and recombinant human Ang-2 were assessed, and (3) a rodent model in which vascular leak was quantified.

## Methods

### Patients

Every weekday during a 2-mo period, all patients admitted during the preceding 24 h to the medical intensive care unit at Beth Israel Deaconess Medical Center were screened for study eligibility. Participants were identified by the parameters used to define sepsis [21]. Evidence of infection (e.g., positive cultures, typical clinical syndrome) must have been accompanied by two of the following four criteria: (1) temperature < 36 °C or > 38 °C; (2) heart rate > 90 bpm; (3) respiratory rate > 20 bpm or PaCO_2_ < 32 mmHg; (4) white blood cell count > 12,000 cells/mm^3^, <4,000 cells/mm^3^, or >10% immature forms. In a prospective fashion, discarded serum samples and clinical data were collected on this cohort as well as from a randomly selected control group of patients hospitalized on the general medical service with a variety of illnesses that did not meet criteria for sepsis. All identifying information was removed; study specimens from patients and clinical data were encoded to protect the privacy of patients. Serum collected from the clinical laboratory was aliquoted in a sterile fashion and stored in cryo-vial tubes at −80 °C. The institutional review board of Beth Israel Deaconess Medical Center approved this study.

### ELISA

Ang-2 levels were measured in serum samples from patients by sandwich ELISA using the reagents and protocol supplied with the human Ang-2 ELISA kit (R&D Systems, Minneapolis, Minnesota, United States). Preliminary experiments confirmed the stability of Ang-2 in serum for 6–12 h at room temperature as well as its stability through several freeze–thaw cycles.

### Chemicals

We purchased human recombinant Ang-1 and Ang-2 from R&D Systems. The RhoA-associated protein kinase inhibitor Y27632 and endothelial cell myosin light chain kinase (EC MLCK) inhibitor ML-7 were purchased from EMD Biosciences (San Diego, California, United States). Other reagents used in the experiments were obtained from Sigma (St. Louis, Missouri, United States).

### Animals

Female FVB mice (obtained from Charles River, Wilmington, Massachusetts, United States) weighing 18–25 g were used throughout the study. All protocols were approved by the Beth Israel Deaconess Medical Center Animal Committee. They were acclimated to the animal facilities for at least 1 wk before the beginning of any experiment.

### Cell Culture

Human microvascular endothelial cells (HMVECs) from neonatal dermis (Cambrex Bio Science, Walkersville, Maryland, United States) were cultured in EBM-2 (Cambrex Bio Science) supplemented with 5% fetal bovine serum (FBS) and growth factors according to the manufacturer's instructions. Serum starvation was performed by incubation in 0.25% FBS/EBM-2 for 24 h.

### Evans Blue Permeability Assay

Mice were injected with 10 μg of Ang-2 or vehicle intraperitoneal, and, after 16 h, were anesthetized with Avertin (2,2,2-Tribromoethanol). 2% Evans blue (50 μl) was then injected into the retro-orbital sinus. (In preliminary experiments with control mice, *n* = 6, we confirmed that the retro-orbital sinus provided a route of intravascular injection that allowed near-100% delivery of Evans blue in a reproducible fashion.) 10 min after Evans blue injection, mice were sacrificed and perfused with PBS with 2mM EDTA for 10 min through a cannula placed in the right ventricle. Blood and PBS were vented through an incision in the vena cava, thus allowing perfusate to pass through the pulmonary and systemic circulations. After 10 min of perfusing the right ventricle with PBS, the outflow from the vena cava was observed to be clear, confirming that blood (and intravascular Evans blue) had been flushed out of the circulation. Washout of intravascular contents was also confirmed histologically after 10 min of PBS perfusion. Organs were then harvested and homogenized in 1.5 ml of formamide. Evans blue was extracted by incubating the samples at 70 °C for 24 h, and the concentration of Evans blue was estimated by dual-wavelength spectrophotometer (620 nm and 740 nm). The following formula was used to correct optical densities (E) for contamination with heme pigments: E620_(corrected)_ = E620_(raw)_ − (1.426 × E740_(raw)_ + 0.030).

### Lung Wet-to-Dry Weight Ratio

Mouse lung wet-to-dry weight ratio (W/D ratio) was used to measure lung water accumulation after Ang-2 injection. Lung wet weight was determined immediately after removal of the right lung. Lung dry weight was determined after the lung had been dried in an oven at 50 °C for 24 h. The W/D ratio was calculated by dividing the wet weight by the dry weight.

### Histology

Adult mice were injected with 0, 10, or 20 μg of Ang-2 intraperitoneal and sacrificed at time zero, 3 h, and 48 h after injection. Lungs were harvested, fixed in 10% formalin, embedded in paraffin, sectioned, and stained with hematoxylin and eosin.

### Western Blot Analysis

Cells were washed with ice-cold PBS three times and lysed with ice-cold RIPA buffer (50 mM Tris-HCl [pH 7.4], 150 mM NaCl, 1% NP-40, 0.5% sodium deoxycholate, 0.1% SDS, and 1 mM EDTA) supplemented with protease inhibitors (Roche Diagnostics, Indianapolis, Indiana, United States) and 1 mM NaF. Lysates were sonicated and centrifuged at 10,000 rpm for 10 min at 4 °C, and supernatants were collected. Protein concentrations were determined by Bradford protein assay with bovine serum albumin as a standard (Bio-Rad, Hercules, California, United States). A mixture of lysate, NuPAGE reducing agent, and NuPAGE sample buffer were heated at 70 °C for 10 min, electrophoresed in NuPAGE 4%–12% Novex Bis-Tris Gels (all from Invitrogen Life Technologies, Franklin Lakes, New Jersey, United States), transferred to polyvinylidene difluoride (PVDF) membrane, and immunoblotted with specific primary antibodies. Binding of primary antibodies was detected using horseradish peroxidase-conjugated secondary antibodies (Amersham Pharmacia Biotech, Piscataway, New Jersey, United States) and SuperSignal WestDura (Pierce Biotechnology, Rockford, Illinois, United States) reagents as chemiluminescence substrates. Primary antibodies were obtained from these suppliers: anti-Tie2 antibody (clone Ab33) was from Upstate Cell Signaling Solutions (Lake Placid, New York, United States); anti-GAPDH monoclonal antibody was from Chemicon International (Temecula, California, United States).

### Immunoprecipitation

For immunoprecipitation of proteins from total cell lysates, we lysed cells with RIPA buffer, adjusting protein concentration by Bradford protein assay (Bio-Rad, Hercules, California, United States) and incubated 200 μg of total protein with anti-Tie2 antibody for 3 h, followed by incubation with protein A sepharose (Zymed, San Francisco, California, United States) for 2 h at 4 °C. After washing the beads, proteins were eluted by heating in SDS-sample buffer and detected by Western blot analysis with Anti-phospho-tyrosine (clone 4G10, Upstate Cell Signaling Solutions, Charlottesville, Virginia, United States) as described before.

### MLC-p

After signal starvation with 0.25% FBS EBM-2 for 24 h, cells were treated with 100 ng/ml Ang-2 or vehicle for 0, 1, 3, and 6 h. MLC-p phospho-serine 19 and GAPDH were detected by Western blot analysis. For MLC-p assays conducted on HMVECs treated with serum from patients, serum Ang-2 concentration was first measured by ELISA. Then, serum was diluted to 5% with EBM-2 and filtered with low-protein-binding PVDF membrane (0.22 μm, Millipore, Bedford, Massachusetts, United States). Anti-MLC-phospho-serine-19 Ab was obtained from Abcam (Cambridge, Massachusetts, United States).

### Rho Activity Pull-Down Assay

RhoA activity assay was performed and quantified using the RhoA activation assay kit according to the manufacturer's instructions (Cytoskeleton, Denver, Colorado, United States). After signal starvation with 0.25% FBS EBM-2 for 24 h, cells were treated with 100 ng/ml Ang-2 or vehicle for the indicated times (0, 30 min, 1 h, 3 h, and 6 h) and harvested into the lysis buffer. Following a brief centrifugation to remove cell debris, lysates from control and Ang-2-treated cells containing equivalent protein concentrations were rotated for 60 min with a 40-μl slurry of a GST-fusion protein composed of the Rho-binding domain of the specific RhoA effector rhotekin coupled to agarose beads. Beads were collected by centrifugation and washed three times with the lysis buffer. Beads were then re-suspended in NuPAGE reducing agent and NuPAGE sample buffer and heated at 70 °C for 10 min. The liquid phase was electrophoresed in NuPAGE 4%–12% Novex Bis-Tris Gels (Invitrogen Life Technologies). Whole-cell lysates from both control and Ang-2-treated cells were also run to determine baseline levels of total RhoA protein. Separated proteins were transferred to nitrocellulose and immunoblotted with a monoclonal antibody to RhoA (Santa Cruz Biotechnology, Santa Cruz, California, United States).

### Immunofluorescence

HMVECs were grown to confluence on glass coverslips coated with 1% gelatin. The cells were fixed for 10 min in 4% paraformaldehyde in PBS, and incubated for 5 min in 0.5% Triton X-100 in PBS. After blocking, the monolayers were processed for staining with anti-VE-cadherin monoclonal antibody (BD Biosciences Pharmingen, San Diego, Califiornia, United States) and Alexa Fluoro 488 goat anti-mouse IgG, rhodamine phalloidin (Molecular Probes, Eugene, Oregon, California) for F-actin staining and TOPRO-3-iodine (Molecular Probes) for nuclear staining. Fluorescence images were obtained using a Bio Rad MRC confocal fluorescence microscope. For experiments using cells treated with serum from patients, serum Ang-2 concentration was first measured by ELISA. Then, patient serum was diluted to 10% with EBM-2 and filtered with low-protein-binding PVDF membrane (0.22 μm, Millipore) prior to application on EC monolayers.

### Measurement of Endothelial Permeability In Vitro

HMVEC monolayer permeability was determined with the use of FITC-labeled bovine serum albumin (Sigma) as described elsewhere [22]. Coster Transwell membranes (Corning, Corning, New York, United States) were coated with fibronectin, and cells were grown until confluence. Vehicle or Ang-2 (400 ng/ml) with FITC-albumin (final concentration 1mg/ml) was added to the luminal chamber for 8 h, and samples were taken from both the luminal and abluminal chamber for fluorometry analysis. The readings were converted with the use of a standard curve to albumin concentration. These concentrations were then used in the following equation to determine the permeability coefficient of albumin (P_a_):



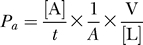



where [A] is abluminal concentration; *t* is time in seconds; *A* is area of membrane in cm^2^; V is volume of abluminal chamber; and [L] is luminal concentration.

### siRNA Transfection of ECs

HMVECs were seeded on 10-cm dishes for Western blot (or on 1% gelatin-coated coverslips for immunohistochemistry experiments) 24 h before experiments. 20 μmol of validated, annealed short interfering RNA (siRNA) (Ambion, Austin, Texas, United States) directed to human Tie-2 was transfected using silentFect Lipid reagent (Bio-Rad) according to the manufacturer's instructions. Cells were used for experiments 3 d after transfection. Down-regulation of Tie-2 receptor was verified by Western blotting with anti-Tie-2 polyclonal antibody (Upstate Cell Signaling Solutions).

### Statistical Analysis

Results are reported as mean ± standard error of the mean. Comparisons between continuous variables were performed using an unpaired two-sided *t*-test.

## Results

### Circulating Ang-2 Is Elevated in Patients with Sepsis Who Have Poor Gas Exchange

Table 1 presents baseline characteristics of the 22 patients with sepsis enrolled in this initial case cohort over a 2-mo period (September 2004 to October 2004). A control cohort of 29 patients was also selected randomly from individuals hospitalized on the general medical service with a variety of illnesses over this same time period. Serum specimens were collected prospectively along with relevant clinical data (see Methods for enrollment criteria, specimen handling, reproducibility of measurements, and specimen storage). Circulating Ang-1 was not different between cases and controls (unpublished data), but Ang-2 at the time of enrollment (Figure 1) was 23.2 ± 9.1 ng/ml among patients with severe sepsis—defined by the presence of shock or multi-organ dysfunction (third bar, *n* = 17)—compared with only 3.5 ± 0.6 ng/ml among non-septic hospitalized controls (first bar, *n* = 29, *p* = 0.0071) and 4.8 ± 1.5 ng/ml among patients with sepsis but who did not have shock or multi-organ dysfunction (second bar, *n* = 5).

**Figure 1:**
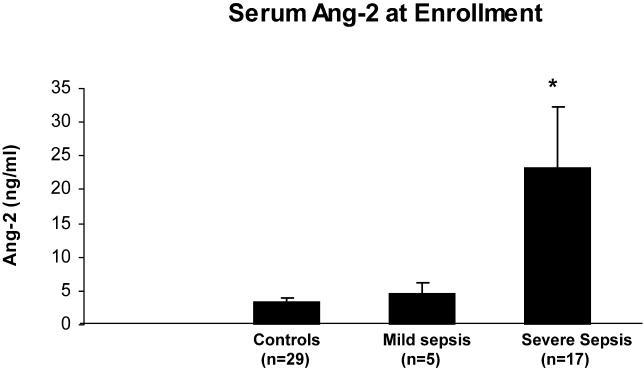
Serum Ang-2 at Study Enrollment Ang-2 was measured in serum specimens obtained prospectively from patients meeting criteria for sepsis (*n* = 22) and from randomly selected hospitalized patients (*n* = 29), used as a control, with a variety of illnesses ranging from infectious (e.g., pyelonephritis, aseptic meningitis, pneumonia) to cardiovascular (e.g., angina, syncope) and neurologic (e.g., stroke) diseases. Patients with sepsis were further subdivided into those with severe sepsis—defined by the presence of shock or multi-organ dysfunction (*n* = 17)—and those without severe sepsis (mild sepsis, *n* = 5). Individuals who were controls (marked Controls) and individuals with sepsis without shock or multi-organ dysfunction (marked Mild Sepsis) had low serum Ang-2 at enrollment (3.5 ± 0.6 ng/ml and 4.8 ± 1.5 ng/ml, respectively). Patients hospitalized with severe sepsis (marked Severe Sepsis) had significantly higher serum Ang-2 at enrollment (23.2 ± 9.1 ng/ml, *p* = 0.0071) compared with control patients. During the course of the hospitalization, only the severe sepsis group had higher peak Ang-2 (32.4 ± 8.7 ng/ml), whereas those patients in the control group and those patients with mild sepsis maintained stable Ang-2 < 10 ng/ml (unpublished data).

**Table 1:**
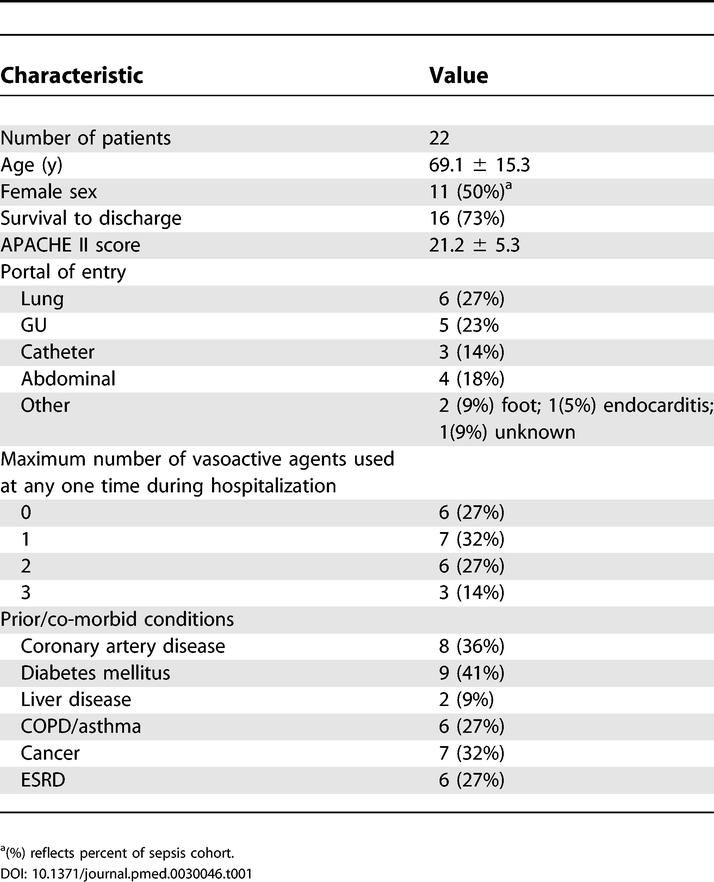
Baseline Characteristics of Sepsis Cohort

Serial measurements of Ang-2 revealed that the group of individuals with severe sepsis—the one that started with the highest circulating Ang-2—developed even higher peak Ang-2 during the course of hospitalization (32.4 ± 8.7 ng/ml), whereas hospitalized patients who did not have sepsis (controls) and patients who had sepsis but were without shock or multi-organ dysfunction (mild sepsis) had Ang-2 serum levels that generally remained ≤10 ng/ml. Our control values for serum Ang-2 concentration are consistent with data reported by others [23].

Serum from Patient CH (Figure 2, —▪—) shows a typical trend of Ang-2 values in a patient with septic shock who subsequently convalesced and was discharged. This patient grew enterococcus from her urine and initially required three vasoactive agents and ventilatory support to manage shock and hypoxemia. The high peak of Ang-2 followed by progressive decline to normal values mirrored the course of her illness. Patient AP's (Figure 2, —▴—) Ang-2 trend was typical for patients who were hospitalized without sepsis. Patient AP was an elderly woman admitted to the general medicine service with an infection, but without evidence of sepsis. On the other hand, Patient AG's (—○—) trend is illustrative of Ang-2 values both during a non-sepsis-related hospitalization (left of break line) as well as a later hospitalization (right of break line) during which the patient died from complications of severe sepsis. Patient AG was first hospitalized for hypotension following excessive fluid removal during dialysis without evidence of infection, systemic inflammatory response, or respiratory compromise (Figure 2, to the left of the broken line, depicted as hospital days 1–3). During this time, his Ang-2 remained flat and <10 ng/ml. However, 3 mo later, he was re-hospitalized for emergent lower leg amputation to treat gangrene and severe sepsis (to the right of the broken line, depicted as hospital days 6–8 for purposes of illustration). This second admission was associated with abruptly rising Ang-2—the last measurement was made on the day of his death despite aggressive measures in the intensive care unit. The rising Ang-2 curve associated with Patient AG's death was typical of patients with severe sepsis who died despite aggressive measures.

**Figure 2:**
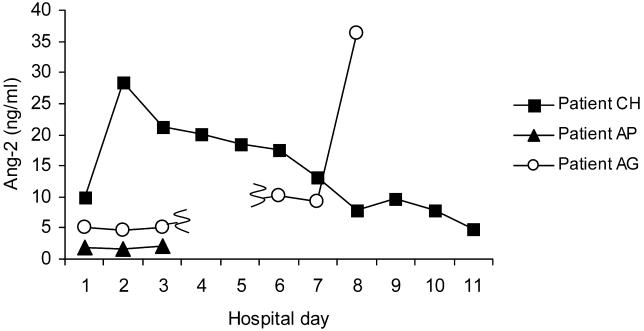
Temporal Trends of Circulating Ang-2 in Three Illustrative Hospitalized Patients Patient CH (—▪—), a 74-y-old woman, was admitted to the medical intensive care unit with severe sepsis. She was treated with broad-spectrum antibiotics, initially required three vasoactive agents to manage shock, and was mechanically ventilated. Patient CH's nadir PaO_2_/FiO_2_ = 240 occurred on hospital day 2, correlating with her peak circulating Ang-2. Enterococcus was grown from her urine. She progressively convalesced and was extubated prior to discharge. Patient AP (—▴—), a 92-y-old woman, was admitted to the general medicine service from a nursing home for increased confusion over her baseline dementia. She had no evidence of sepsis, shock, or respiratory compromise—PaO_2_/FiO_2_ > 300. She was treated for a foot wound infection with two antibiotics and was discharged in stable condition back to the nursing home. Patient AG (—○—), a 77-y-old man, was first admitted to the general medicine service with hypotension following excessive fluid removal at hemodialysis—there was no evidence of infection, systemic inflammatory response, or respiratory compromise with PaO_2_/FiO_2_ > 300 (hospital days 1–3). However, 3 mo later (graphed as hospital days 6–8 for purposes of illustration), the same patient (—○—) was re-admitted to the intensive care unit following emergent right leg amputation for gangrene complicated by shock and inability to extubate. Nadir PaO_2_/FiO_2_ = 144 occurred on the same day as peak Ang-2 (depicted as hospital day 8), when he died despite full care.

Given our hypothesis that Ang-2 imbalance should preferentially affect lung permeability—where Tie-2 expression is highest [19]—we compared peak circulating Ang-2 value during hospitalization between individuals with very poor oxygenation versus those with less impaired oxygenation. PaO_2_/FiO_2_, the ratio of arterial blood's oxygen partial pressure (PaO_2_) to the fraction of inspired air consisting of oxygen (FiO_2_) [24], was used as a metric to assess the defect in oxygen absorption from the lung into the bloodstream. A cutoff of 200 in the PaO_2_/FiO_2_ ratio was used to segregate groups into more and less severe impairment in gas exchange because it is one of the diagnostic criteria for ARDS [5]. Indeed, Ang-2 did correlate strongly with nadir impairment in gas exchange—41.95 ± 12.44 ng/ml among those patients with PaO_2_/FiO_2_ < 200 versus 11.22 ± 2.44 ng/ml in the group of individuals with better oxygenation (PaO_2_/FiO_2_ > 200); *p* = 0.02 (Figure 3A). Ang-2 did not correlate with survival to discharge nor to level of critical illness at enrollment as defined by APACHE II (acute physiology and chronic health evaluation) (Figure 3B and 3C). Ang-2 also did not correlate with a history of congestive heart failure (Figure 3D). There was also no correlation of Ang-2 with an underlying history of lung disease, renal failure, or cancer (unpublished data). Of note, the one patient in our cohort who did meet full criteria for ARDS had Ang-2 = 139 ng/ml, the highest level measured in our group. Circulating Ang-2 elevation, therefore, appeared to correlate specifically with a low PaO_2_/FiO_2_ ratio.

**Figure 3:**
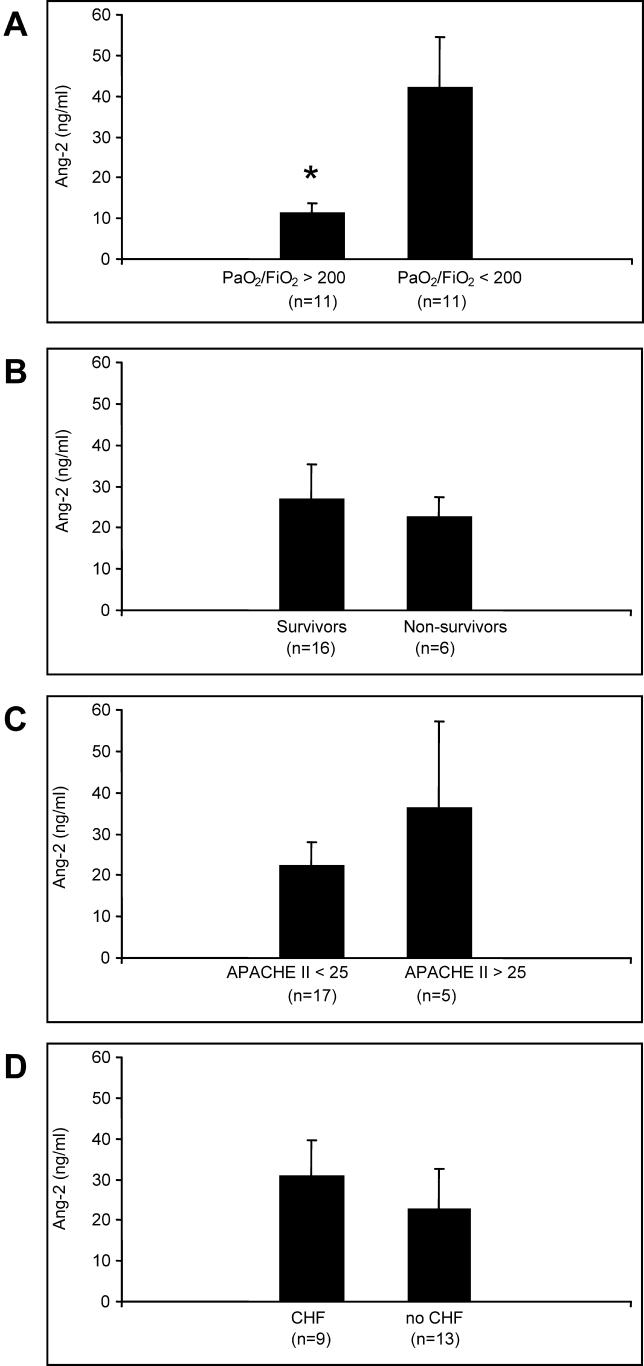
Peak Circulating Ang-2 Correlates with Impaired Pulmonary Gas Exchange (A) Impaired oxygenation of blood, as assessed by the nadir PaO_2_/FiO_2_ ratio, correlates with significant differences in circulating Ang-2, *p = 0.0195. (B) Circulating Ang-2 does not correlate with survival to discharge. Among the five patients who did not survive, medical care was withdrawn from three patients in accordance with family wishes; the remaining two died despite full measures. (C) APACHE II is a commonly used scoring system to rate overall severity of critical illness. Ang-2 does not differ significantly among individuals with high (more severe illness) or low (less severe illness) APACHE II scores. (D) History of congestive heart failure (defined by clinical documentation in medical record of measured ejection fraction = < 40%) does not correlate with significant differences in circulating Ang-2.

### Serum from Patients with Sepsis Disrupts Endothelial Architecture, an Effect that Resolves with Clinical Convalescence, Correlates with Ang-2, and Is Reversed by Ang-1

Separation of adjacent ECs from one another leads to paracellular gap formation—a process driven by actin–myosin-based cell contraction [25–27]. Such gaps permit para-endothelial movement of macromolecules and, thus, represent a structural change that correlates with hyperpermeability. To test what structural effect human serum had on cultured ECs, we added serum from two patients, Patient CE4 (high Ang-2) and Patient CF1 (low Ang-2), to HMVECs and stained for F-actin and VE-cadherin, a structural protein that helps maintain intercellular junctions. Incubation of HMVECs with control medium (FBS/culture medium) resulted in a compact, confluent cell layer with thin actin filaments and localization of VE-cadherin to cell–cell junctions (Figure 4A–4C). However, addition of high Ang-2 serum (CE4, Ang-2 = 89 ng/ml) induced thick actin stress fibers and intercellular gap formation (Figure 4D–4F, arrows) whereas low Ang-2 serum (CF1, Ang-2 = 8.9 ng/ml) did not (Figure 4G–4I). The gap formation provoked by the serum from Patient CE4 was reversed with addition of recombinant human Ang-1 (Figure 4J–4L).

**Figure 4:**
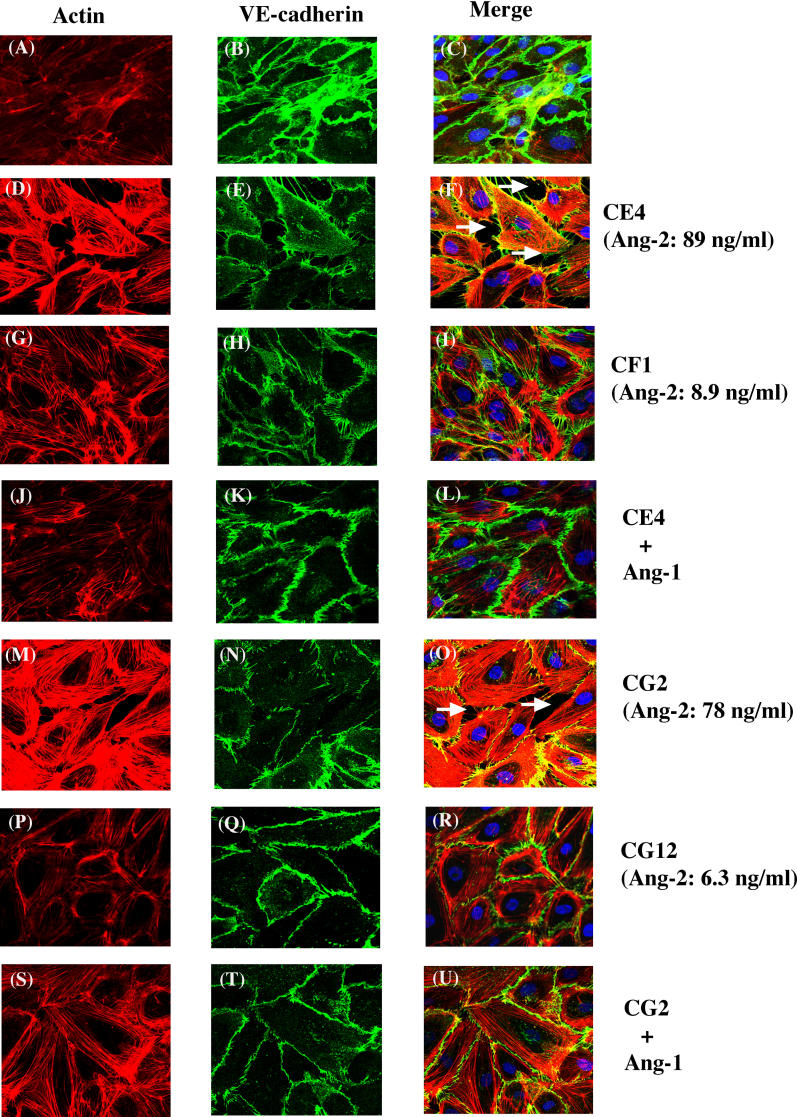
Serum from People with Sepsis Disrupts Endothelial Architecture and This Effect Resolves with Clinical Improvement, Correlates with Measured Ang-2, and Is Reversed by Ang-1 Ten percent FBS or 10% serum from one of two patients with sepsis was incubated with EC monolayers to assess effects on endothelial architecture. High Ang-2 serum (Patient CE4, Ang-2 = 89 ng/ml) induced thick actin stress fibers and intercellular gap formation (D–F), whereas low Ang-2 serum (CF1, Ang-2 = 8.9 ng/ml) did not (G–I). The gap-promoting effect of Patient CE4′s serum was reversed with addition of 100 ng/ml recombinant human Ang-1 (J–L) and was indistinguishable from control cells that exhibit thin actin fibers and no intercellular gaps (A–C). Serum was then taken from one patient (Patient CG), drawn on hospital day 2 (Patient CG2, Ang-2 = 78 ng/ml) and hospital day 16 (Patient CG12, Ang-2 = 6.3 ng/ml), and was added at 10% to HMVEC monolayers. Again, high-Ang-2 serum (CG2) induced gap formation and thick actin stress fibers (M–O), effects not seen in the serum of the same patient at discharge (CG12) (P–R) and effects that were reversed with the addition of 100 ng/ml Ang-1 (S–U). Arrows indicate intercellular gaps.

To address the potential biasing effect of unmeasured confounders between sera from two different patients, we repeated this experiment with serum from one patient taken at two time points during his hospitalization. Serum from Patient CG was collected on hospital day 2 (CG2, Ang-2 = 78 ng/ml), and on hospital day 16 (CG12, Ang-2 = 6.3 ng/ml). On hospital day 2, Patient CG had PaO_2_/FiO_2_ = 56, was in septic shock, and had ARDS; by hospital day 16, Patient CG was extubated, convalescing uneventfully and preparing for discharge. Serum from CG's hospital day 2 (Figure 4, CG2) induced gap formation and thick actin stress fibers (Figure 4M–4O), effects not seen with his serum at discharge (Figure 4P–4R, CG12); moreover, effects of high Ang-2 serum from hospital day 2 (Figure 4, CG2) were reversed with the addition of Ang-1 (Figure 4S–4U).

These results illustrate (a) the presence of a serum activity during severe sepsis that induces endothelial barrier disruption; (b) that clinical resolution correlates with falling Ang-2 and decreased barrier-disrupting activity; and (c) that this activity can be reversed with Ang-1, suggesting that Ang-2 in the serum of human patients is at least partially responsible for altering endothelial architecture in sepsis.

### Ang-2 Alone Replicates the Effect of Serum from Patients with Sepsis on Endothelial Structure and Promotes Endothelial Hyperpermeability

Having observed this effect of serum from human patients on cultured ECs, we next tested whether Ang-2 alone could reproduce disruption of endothelial architecture. Recombinant human Ang-2 (100 ng/ml) was added to HMVECs, which were subsequently stained for F-actin and VE-cadherin. As suspected, Ang-2 induced the formation of thick actin stress fibers and intercellular gaps (Figure 5Ad–Figure 5Af, arrows), effects not seen with vehicle incubation (Figure 5Aa–Figure 5Ac). This experiment confirmed the hypothesis raised by the results in Figure 4—namely that Ang-2 alone could provoke potentially pathologic structural changes in endothelium.

**Figure 5:**
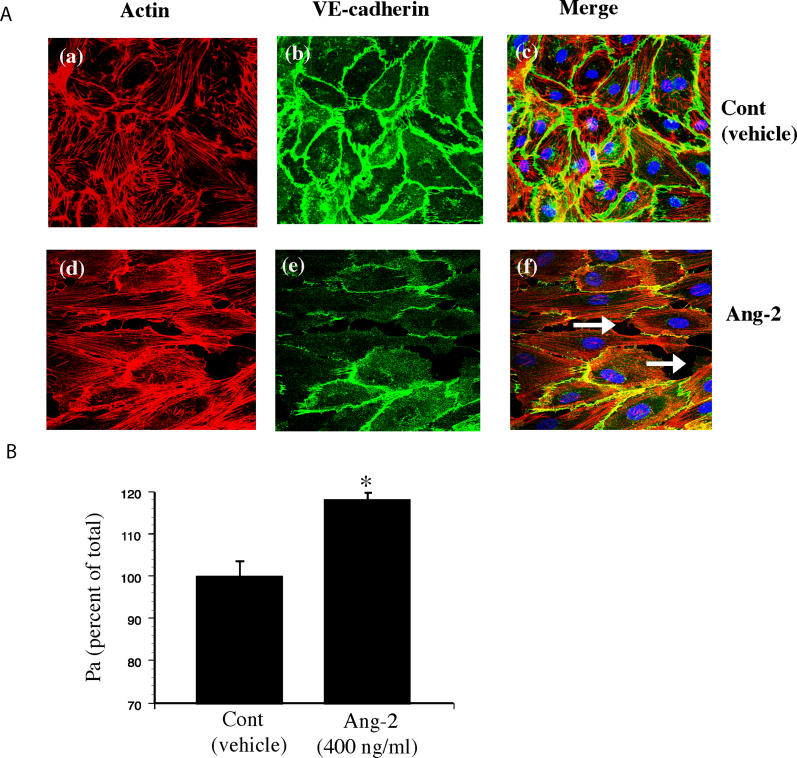
Ang-2 Alone Disrupts Endothelial Architecture at Physiologic Concentrations (A) Control (vehicle) or recombinant human Ang-2 (100 ng/ml) was added to HMVEC monolayers. These cells were then fixed and stained for F-actin and VE-cadherin. Shown are healthy control cells (panels a–c) versus Ang-2 treated cells (panels d–f), which exhibit thick actin stress fibers and disrupted junctions, leaving intercellular gaps (arrows). (B) HMVECs were grown to confluence on Transwell membranes coated with fibronectin. Monolayers were treated with vehicle or Ang-2 (400 ng/ml in luminal chamber) plus FITC-albumin. P_a_ was calculated after 8 h as described in the Methods section. P_a_ values are expressed as percentage of control cells.*p < 0.01.

After establishing that Ang-2 could promote pathologic structural changes in endothelial monolayers, we next asked whether Ang-2 could modulate barrier function. To determine the effect of Ang-2 on monolayer permeability, we monitored the clearance of FITC-labeled albumin across an HMVEC monolayer with and without Ang-2 stimulation. Figure 5B shows that Ang-2 stimulation for 8 h increased permeability by approximately 20% compared with the control (*p* < 0.01).

### The Effect of Ang-2 on Endothelial Architecture Is Mediated by Rho-Kinase and MLC-p

Since Ang-2 appeared to be a likely mediator of endothelial barrier disruption in serum from humans with sepsis, we next sought to understand the intracellular mechanism through which Ang-2 could distort endothelial shape and cell–cell contacts.

Endothelial barrier function is known to be tightly regulated by myosin-driven cellular contraction [8,28–30]. For contraction to occur, MLC must be phosphorylated at Ser-19 by EC MLCK, and phosphorylation of Ser-19 by EC MLCK is needed to activate actomyosin ATPase function [31,32]. We therefore hypothesized that Ang-2 up-regulated MLC-p.

Initially, we tested the effect of serum from humans with sepsis on MLC-p. Serum was taken from the same patients used for immunohistochemistry in Figure 4D–4I—CE2 (Ang-2 = 77 ng/ml) and CF5 (Ang-2 = 7.9 ng/ml)—and added at 1:20 dilution to 24-h serum-starved HMVECs. The high Ang-2 serum (CE2) caused MLC-p that was inhibited by addition of Ang-1, whereas the low Ang-2 serum led to markedly less MLC-p (Figure 6A).

**Figure 6:**
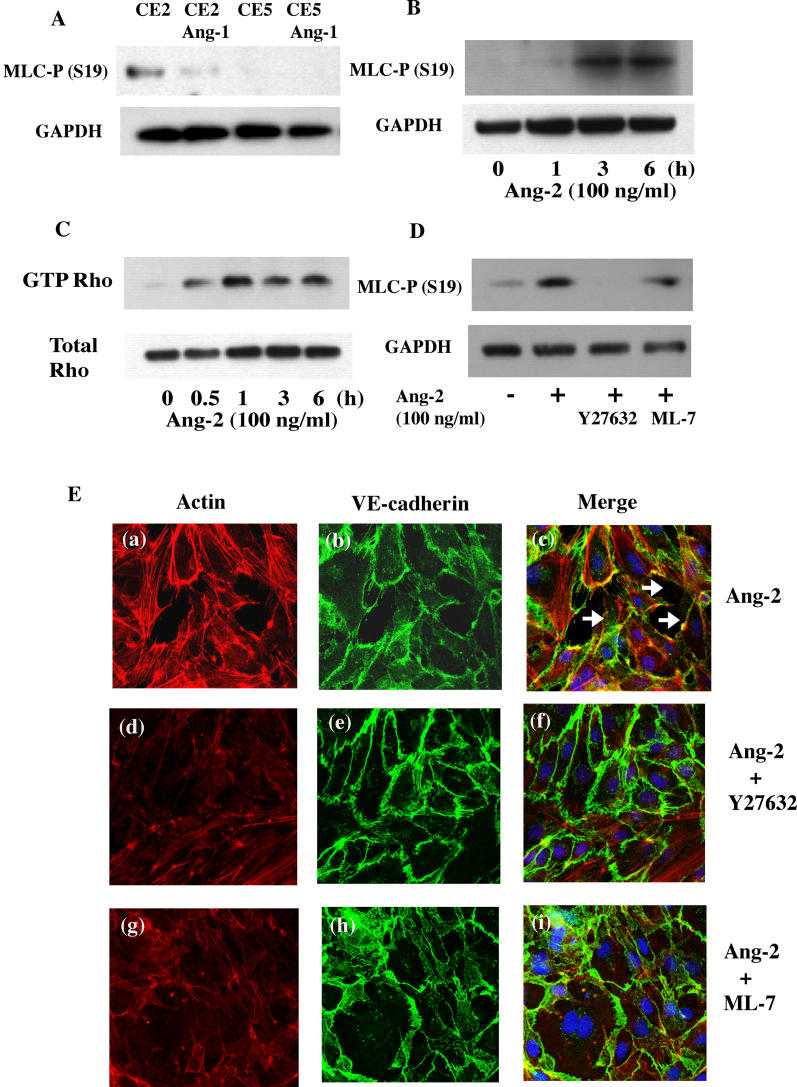
Ang-2 Disrupts Endothelial Architecture by Upregulating MLC-p in an MLCK- and Rho-Kinase–Dependent Fashion (A) Serum was taken from two patients—Patient CE2 (Ang-2 = 77 ng/ml) and Patient CF5 (Ang-2 = 7.9 ng/ml)—and added at 20-fold dilution to 24-h serum-starved HMVECs. High Ang-2-serum (Patient CE2) caused MLC phosphorylation that was diminished by addition of Ang-1 (100 ng/ml), whereas low Ang-2-serum (CF5) did not induce MLC phosphorylation. (B) After 24-h serum starvation, Ang-2 (100 ng/ml) was added to HMVECs, and cells were lysed at the indicated times. MLC-p was determined by Western blot as described in the Methods section. MLC-p was elevated at 3 h and 6 h of stimulation. (C) After 24-h serum starvation, Ang-2 (100 ng/ml) was added to HMVECs, and cells were lysed at the indicated times. GTP-RhoA was pulled down and blotted as described in the Methods section. GTP-RhoA peaked at 30–60 min of Ang-2 stimulation. (D) After 24-h serum starvation, HMVECs were stimulated with Ang-2 (100 ng/ml) with or without 10 μM Y27632 (Rho-kinase inhibitor) or 10 μM ML-7 (MLCK inhibitor) for 5 h. MLC-p was determined by Western blot as described in the Methods section. Y27632 had a more potent inhibitory effect on MLC-p than equimolar ML-7. (E) HMVECs were grown to confluence and incubated for 5 h with Ang-2 (100 ng/ml) (panels a–c). HMVECs were also stimulated with Ang-2 (100 ng/ml) in the presence of 10 μM Y27632 (panels d–f) or 10 μM ML-7 (panels g–i). Cells were fixed and stained for F-actin and VE-cadherin as described in the Methods section. Shown are representative confocal fluorescence microscopy images (600×). Ang-2 provokes stress fibers within cells (panel a) and gap formation between cells (panel c, arrows). These changes are reversed by co-incubation with Y27632 or ML-7. F-actin, panels a, d, and g; VE-cadherin, panels b, e, and h; merge images, panels c, f, and i.

Next, we tested the effect of Ang-2 alone (100 ng/ml) on 24-h serum-starved HMVECs at serial time points. We observed that MLC-p was elevated at 3 h and 6 h of stimulation (Figure 6B) and persisted for 24 h (unpublished data).

Rho-GTPases play a pivotal role in the control of cellular actin rearrangement and cell shape [33]. Rho-kinase, a downstream target of RhoA, stimulates stress fiber formation by up-regulating MLC-p through two mechanisms: activation of EC MLCK and inhibition of myosin phosphatase activity [34,35]. Given this 2-fold effect of RhoA on MLC, one would predict that a RhoA inhibitor would be even more potent than an MLCK inhibitor at blocking MLC-p. We asked if Ang-2-induced MLC-p required activated RhoA. We tested this possibility by treating 24-h-serum-starved HMVECs with Ang-2 (100 ng/ml) for different time periods (0, 30 min, 1 h, 3 h, and 6 h) and then by measuring RhoA activation. Indeed, Ang-2 induced an increase in the active form of RhoA (Rho-GTP) (Figure 6C) that peaked between 30 min and 1 h. Pre-treatment of Ang-2-stimulated HMVECs with a specific inhibitor of Rho-kinase (Y27632, 10 μM) completely abolished Ang-2-induced MLC-p (Figure 6D, third column) while an EC MLCK inhibitor (ML-7, 10μM) partially inhibited MLC-p (Figure 6D, fourth column). These results demonstrate that Ang-2 mediates MLC-p in a RhoA-dependent fashion in human microvascular ECs.

Given that Ang-2 induces gap formation between ECs (Figure 5) and that Ang-2 stimulates MLC-p through Rho-kinase (Figure 6B–6D), we next asked whether Ang-2′s gap-formation effect occurred through Rho-kinase and MLCK. Y27632 (10 μM) completely reversed the formation of thick actin stress fibers and paracellular gaps induced by Ang-2 (Figure 6Ea–Figure 6Ef). ML-7 (10 μM) partially reversed the Ang-2-induced structural changes on actin and cell junctions (Figure 6Eg–Figure 6Ei), consistent with the less potent effect of ML-7 versus Y27632 on MLC phosphorylation (Figure 6D). These results demonstrate that the deleterious structural effects of Ang-2 on ECs are mediated through Rho-kinase and MLCK.

### Tie-2 Knock-Down Replicates the Ang-2-Induced Endothelial Barrier Disruption

Multiple lines of evidence suggest that Ang-1 and Ang-2 are an agonist–antagonist pair at the Tie-2 receptor [36]. Ang-1 activates Tie-2, leading to receptor phosphorylation and subsequent signal transduction that promotes EC survival and vessel assembly. Ang-2, on the other hand, is believed to act as a Tie-2 ligand that competitively binds the receptor and interferes with agonistic Ang-1/Tie-2 functions—i.e., Ang-2 binding of Tie-2 blocks Tie-2 phosphorylation. To further delineate the pathway through which Ang-2 promotes EC contraction and gap formation, we first confirmed that Ang-2 (100 ng/ml) inhibited phosphorylation of Tie-2 receptor in HMVECs (Figure 7A).

**Figure 7:**
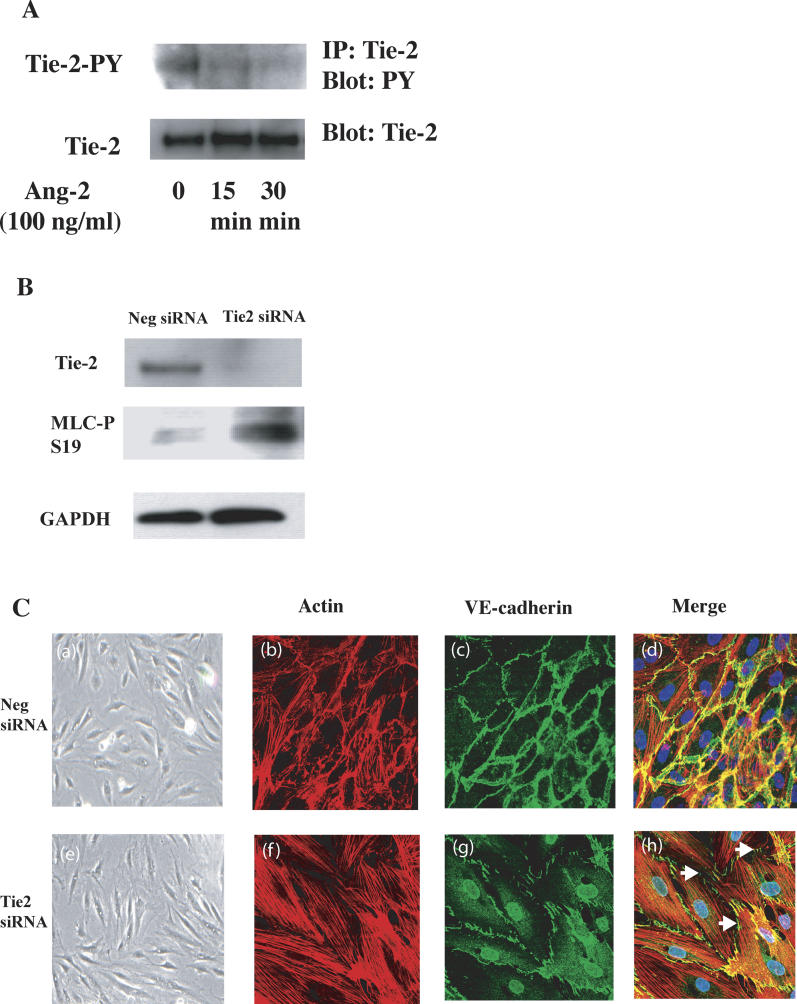
Tie-2 Knock-Down Inhibits MLC-p and Promotes Disrupted Endothelial Architecture, Replicating the Effects of Ang-2 (A) HMVECs were stimulated with Ang-2 (100 ng/ml) in 2.5% FBS EBM-2 for the indicated times, and phospho-Tie-2 was detected by immunoprecipitation and Western blot (upper) as described in Methods. Similar amounts of total Tie-2 were present in HMVECs harvested at each time point. Phospho-Tie-2 declined over time whereas total Tie-2 remained relatively constant (lower). (B) Negative control siRNA (left column) or a Tie-2-specific siRNA (right column) was transfected in HMVECs. Cells were then serum-starved for 24 h, after which decreased Tie-2 expression (right column, upper blot) and increased MLC phosphorylation (right column, middle blot) were verified with Tie-2 siRNA. (C) Phase contrast (200×) and fluorescence images (600×) of cells stained for F-actin and VE-cadherin after transfection of negative control siRNA (panels a–d) or Tie-2 specific siRNA (panels e–h). Tie-2-siRNA caused thick actin stress fibers and gap formation in HMVECs (panel h, arrows). Phase contrast images, panels a and e; F-actin, panels b and f; VE-cadherin, panels c and g; merge images, panels d and h.

We then assessed the effect of Tie-2 signaling on MLC-p by using siRNA against Tie-2 receptor (Tie-2-siRNA). Tie-2-siRNA induced robust MLC-p (Figure 7B, middle blot), recalling the effect seen with Ang-2 treatment (Figure 6B). Tie-2-siRNA caused a spindle phenotype (Figure 7Ce), thick actin stress fibers and paracellular gap formation (Figure 7Cf–Figure 7Ch, arrows), effects not observed with negative control siRNA transfection (Figure 7Ca–Figure 7Cd). These morphologic changes are on the same spectrum as, but even more severe than, those seen with the addition of Ang-2 (Figure 5Ad–Figure 5Af). These results suggest that Tie-2 signaling is constitutively active in this system. Addition of Ang-2 blocks Tie-2 signaling, leading, in turn, to Rho-kinase activation and MLC-p, with the end result being EC contraction, gap formation, and disruption of barrier integrity.

### Systemic Administration of Ang-2 Provokes Vascular Hyperpermeability and Pulmonary Congestion in Healthy Adult Mice

So far, we had identified Ang-2 as an important circulating factor in serum from humans with sepsis that distorts the endothelial barrier, and we had determined that this action was carried out by Tie-2 blockade and activation of Rho-kinase and MLCK. To establish the functional importance of maintaining this barrier, we hypothesized that systemic administration of Ang-2 would provoke pulmonary vascular hyperpermeability and congestion.

Evans blue avidly binds to serum albumin and can therefore be used as a tracer for trans-capillary flux of macromolecules. The extravasation of Evans blue has frequently been employed to quantify in vivo vascular permeability [37,38]. Given the several limitations of in vitro permeability assays—e.g., lack of flow and variable hydrostatic pressure, use of cultured cells, absence of microenvironment, and absence of interacting cell types such as neutrophils [39]—we felt that it was important to confirm leak across intact blood vessels in an in vivo setting. Adult mice were pre-treated with either vehicle or Ang-2 (10 μg) injected intraperitoneally prior to Evans blue dye injection. Although no mice in either group exhibited any signs of sickness (e.g., motionlessness, piloerection, diarrhea) 16 h after treatment, spectrophotometric quantification of extravasated dye showed enhanced leakage, with a 3-fold increase in lungs and a 2-fold increase in livers of Ang-2-treated mice compared with vehicle-treated mice (Figure 8A) (*p* < 0.01). Intestines were also tested and showed a statistically non-significant trend toward increased permeability (Figure 8A).

**Figure 8:**
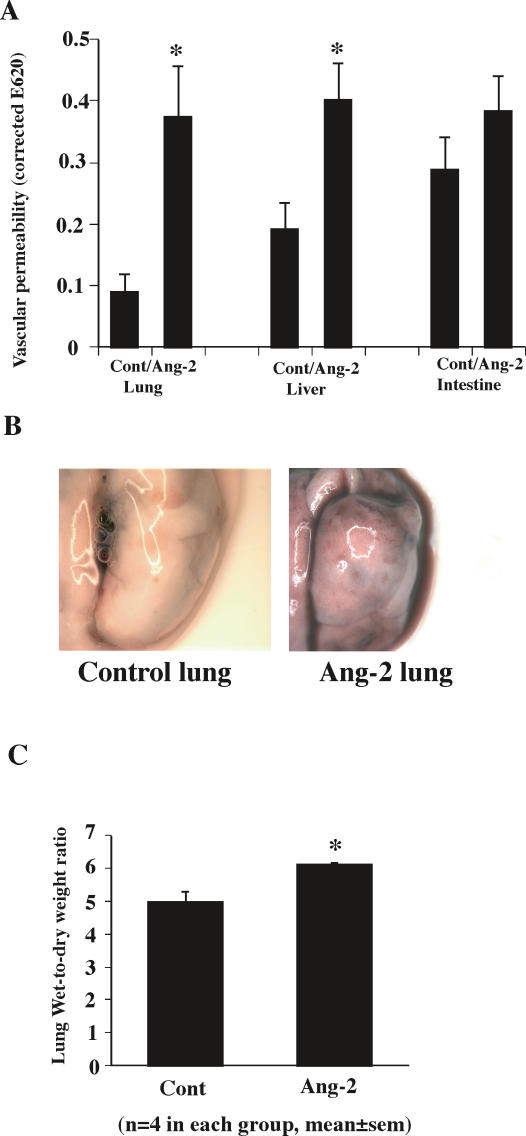
Systemic Administration of Ang-2 Promotes Pulmonary Hyperpermeability and Water Accumulation (A) After injection of vehicle or Ang-2 (10 μg, intraperitoneal), mice were injected in the retro-orbital sinus with Evans blue (2%, 50 μl); after sacrifice, intravascular Evans blue was washed out with PBS and vascular leakage was evaluated by measuring extravasated Evans blue. The amount of Evans blue in organ homogenates was spectrophotometrically quantified. Evans blue content significantly increased in the lung and liver of Ang-2–treated mice, indicating leakage out of the vasculature and impregnation within the tissue, *p < 0.01. (B) Representative photographs of lungs were taken after washout of intravascular Evans blue with PBS for 10 min. The lung from a control (vehicle intraperitoneal) mouse (left) appears blanched in contrast to the purple-tinted, congested lung from an Ang-2-treated mouse (right). (C) The lung W/D weight ratio was determined as described in the Methods section. Ang-2 treatment for 16 h increased lung W/D weight ratio, consistent with congestion due to water accumulation, *p < 0.01.

After washout of intravascular Evans blue by perfusing PBS through the right ventricle and venting from the vena cava, lungs of vehicle-treated mice were blanched-appearing (Figure 8B, left panel); however, lungs of Ang-2-treated mice appeared more congested and purple-tinted (Figure 8B, right panel), suggesting retention of dye in the extravascular space. In addition, the lung W/D weight ratio increased from 5.01 ± 0.26 to 6.13 ± 0.03 with Ang-2 treatment (Figure 8C) (*p* < 0.01), suggesting increased lung water accumulation following Ang-2 administration.

Lung sections were also taken for histologic characterization. Figure 9A shows lung from a control mouse injected with vehicle—alveolar septa form a fine, thin network (inset on lower right). 3 h after systemic Ang-2 administration (10 μg), there is an increase in cellularity, congestion, and early extravasation into air spaces (Figure 9B). These changes are even more pronounced at 48 h (Figure 9C) following a total Ang-2 dose of 20 μg. These results establish an in vivo causative role for Ang-2 in pulmonary vascular leak and further substantiate the in vitro permeability effects observed earlier with Ang-2 stimulation (Figure 5). Moreover, progressive lung injury appears to occur with increasing amount and duration of systemic Ang-2 exposure.

**Figure 9:**
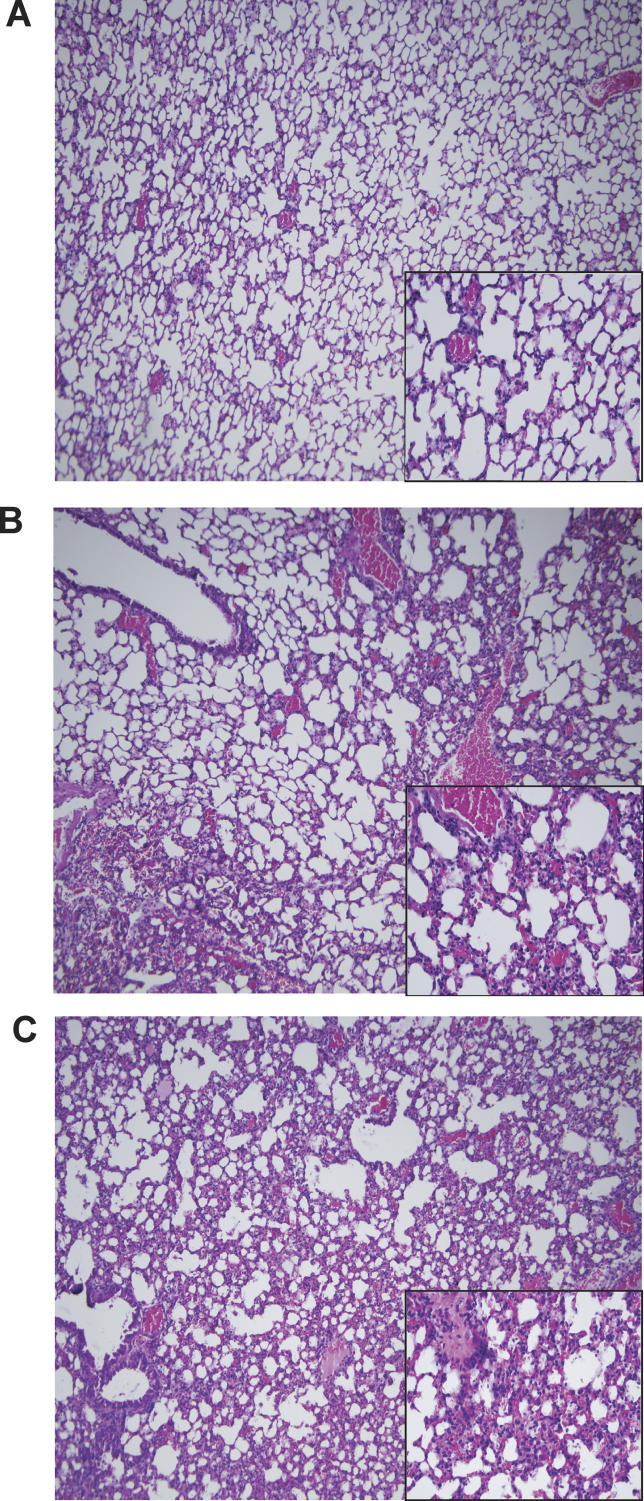
Systemic Ang-2 Administration Provokes Rapid and Progressive Pulmonary Congestion Ang-2 was administered intraperitoneally (10 μg), and lung sections were assessed for histologic changes. Control lung is shown at 100× in (A). Note the thin alveolar septa, particularly in the inset (400×). (B) 3 h after Ang-2, there is noticeable expansion of alveolar septa with increase in cellularity, reduction in air space, and some leakage of cells into the alveolar space. (C) These changes are more advanced after 2 d of systemic Ang-2 administration (total dose 20 μg).

## Discussion

Our results support the hypothesis that Ang-2 is a mediator of pathologic vascular leak in the lung. We show that Ang-2 elevation in patients with sepsis correlates with impaired oxygenation. We demonstrate that the ability of serum from humans with sepsis to disrupt endothelial architecture correlates with Ang-2 level, improves with clinical convalescence, and is reversed by Ang-1. We show that Ang-2 is not simply a marker in severe sepsis, but that it has a causative role in disturbing endothelial architecture. We elucidate this causal role for Ang-2 by identifying an intracellular mechanism linking Ang-2 to MLC-p. Last, we administer Ang-2 to healthy mice and induce severe pulmonary vascular leak and congestion. These results collectively argue that elevated circulating Ang-2 occurs in severe sepsis and that excess Ang-2 can produce pulmonary hyperpermeability in vivo.

Vascular leak has been difficult to assess routinely in the clinical setting because there are no widely applicable tools to measure this process—imaging of extravasated radiolabeled macromolecules is primarily a research tool [40–44]. Despite this limitation, endothelial hyperpermeability has been identified as an important early pathophysiologic contributor to the development of ARDS and acute lung injury [24]. The particular susceptibility of the lung to vascular leak has not been adequately understood. From a purely physiological standpoint, the low hydrostatic pressures in the pulmonary vascular tree should actually mitigate fluid extravasation. However, given that Tie-2 mRNA and protein are most abundant in the lung [19,20], we believe that the lung is uniquely dependent on Tie-2 signaling to maintain the permeability barrier, and that sepsis provides a model system in which that signaling is blocked by excess circulating Ang-2.

Though excess systemic Ang-2 provokes extravasation from and congestion of the pulmonary capillary bed, we have not definitively shown that this effect is due only to endothelial changes. In fact, an intriguing possibility is that excess Ang-2 both distorts microvascular architecture—a conclusion that can be inferred from our data—but also potentially interferes with larger vessels by exerting effects on tone that change hydrostatic pressure to favor extravasation. Such an effect on tone could occur through endothelial-dependent signaling—e.g., nitric oxide [45]—or perhaps through direct effects on smooth muscle itself. For example, it is unknown if smooth-muscle-lined vessels express “endothelial-restricted” receptors such as Tie-2 in stress states such as sepsis. Such suggested effects of Ang-2 on muscle cells may even occur in a Tie-2-independent fashion, as one recent report asserts [46].

Our data point to *activation* of a signaling system (Rho-kinase, EC MLCK) upon *withdrawal* of Tie-2 phosphorylation. The likeliest explanations for this observation are either (a) that the activated receptor is sequestering an activator of the Rho-kinase pathway or (b) that the activated receptor up-regulates an inhibitor of the Rho-kinase pathway. We are exploring several candidate mediators that meet these criteria to bridge this signaling gap. Though the entire pathway remains to be mapped, there are immediate therapeutic implications of the data presented here.

MLC-p may represent a final common pathway for multiple provocateurs of vascular leak—TNF-α, IL-1, complement components—to exert their pathologic pro-permeability effect [47–49]. Inhibitors along this pathway, such as a RhoA inhibitor, would be predicted to block vascular leak. Blockade of one downstream target of RhoA, EC MLCK (by conditional knock-out and chemical inhibition), has already been shown to protect mice from endotoxin-mediated lung injury, further bolstering this hypothesis [30].

Inhibition of EC MLCK may allow clinicians to treat poor barrier function without direct immunomodulation, as opposed to blockade of TNF-α, IL-1, or complement proteins, all of which are interventions that also affect innate and/or adaptive immunity. Undesired pleiotropic effects, such as poorly timed immunosuppression, may be one reason why these therapies have failed in clinical trials for sepsis. Theoretical advantages of directing therapy to Tie-2 to prevent, stabilize, or reverse leak conditions such as sepsis are the lack of global immunosuppression and the expected restriction of effects to the vasculature, particularly to the pulmonary vasculature where Tie-2 is most abundantly expressed. This remains a hypothesis until a clinical trial demonstrates the benefit in sepsis/ARDS, but one report has shown a beneficial effect of Tie-2 agonism in a mouse endotoxin model of shock [50]; another study has shown that locally elevated Ang-2 can provoke extravasation in the mouse hind paw [51]. These results independently affirm the notion of an Ang-1–Ang-2 toggle that tips in favor of the latter to promote vascular leak.

The clinical data presented in Figures 1–3 come from a pilot cohort of patients with sepsis. Undoubtedly, a larger study is needed, particularly one that enrolls ARDS patients who do not have sepsis, to test whether Ang-2 is a valid biomarker for diagnosis, stratification, or prognosis in this condition. However, despite the small sample size and the relative wellness of the patients we studied, we were still able to identify a robust correlation between very poor PaO_2_/FiO_2_ ratio and Ang-2 elevation. We observed that peak Ang-2 did occur at the same time as nadir PaO_2_/FiO_2_ ratio, and that peak Ang-2 followed the most severe stage of illness by ∼24 h (unpublished data). The delayed rise in Ang-2 following the most severe stage of illness may imply that an angiopoietin-signaling disturbance occurs as a secondary phenomenon to release of early-phase cytokines such as TNF-α. But, given that ARDS due to non-pulmonary causes also classically occurs as a delayed phenomenon, the later rise in Ang-2 may help explain why the delay in extra-pulmonary ARDS onset exists.

If Ang-2 is confirmed as a relevant biomarker of acute lung injury, then it may help to determine prognosis and to stratify patients, especially in clinical trials to treat ARDS. Given the rapidity with which Ang-2 rises in severe sepsis (Figure 2, Patient AG, —○—), it is less likely to be an effective screening tool if tested only one time several days before onset of permeability edema in the lungs. On the other hand, if serial Ang-2 values are followed in at-risk patients, then a rising slope could identify someone who would particularly benefit from therapy directed against leak itself in an effort to halt the evolution of ARDS at its earliest exudative phase.

Our results must be interpreted with caution. We have shown a correlation between serum Ang-2 and poor gas exchange in patients with severe sepsis. The correlation may not be causative, and several alternate possibilities must be ruled out in future investigations. First, based on the anti-leakage effect of Ang-1 on mature vasculature [17,52] and the pro-leakage phenotype we have observed with excess Ang-2, we believe Ang-2 is acting as a Tie-2 antagonist in the setting of sepsis as per its initial description [12], but it is worth remembering that the action of Ang-2 on Tie-2 appears to be dose-, duration-, and context-specific in vitro [12,53,54]. Second, Ang-2 release can be driven by inflammatory stimuli such as TNF-α that are clearly present in sepsis, and, therefore, Ang-2 elevation may represent only an epiphenomenon without functional significance—this is less likely as our results demonstrate actual pathologic effects, in vitro and in vivo, of excess Ang-2 alone.

Several additional questions remain to be answered in future studies. First, as mentioned, Ang-2 is a known hypoxia-induced gene product [55–57], but its source(s) in sepsis are unclear. A study of critically ill patients with hypoxia but without sepsis, such as those with cardiogenic shock, would help elucidate whether hypoxia alone induces pathologic elevation in circulating Ang-2.

Second, Ang-2 expression is also up-regulated by vascular endothelial growth factor (VEGF) and basic fibroblast growth factor [57]. Interestingly, serum VEGF appears to be elevated in severe sepsis [58]. VEGF is known to be a pro-permeability ligand, but it may be that Ang-2 partially mediates VEGF's permeability effect; alternatively, VEGF and Ang-2 may be acting in parallel to provoke vascular leak. However, VEGF blockade alone did not improve survival in one report studying a well-accepted rodent model of sepsis [59]. Our results suggest that excess Ang-2 is sufficient to promote pulmonary vascular leak and congestion, but pending experiments to block Ang-2 action in sepsis/ARDS models will help assess the relative contributions of VEGF and Ang-2 to sepsis/ARDS more definitively.

Third, pre-formed Ang-2 can be released from ECs stimulated by inflammatory ligands, such as TNF-α [60], or even released non-specifically following EC death. What remains to be determined is what cell type(s) and what organ(s) account for the elevated Ang-2 level. Apoptosis alone is unlikely to account for Ang-2 elevation for two reasons: (a) under conditions in which only limited apoptosis is observed following in vitro stimulation of ECs with endotoxin, significant up-regulation of Ang-2 is noted (unpublished data); moreover, (b) endotoxin stimulation in vivo also appears to result in organ-specific up-regulation of Ang-2 transcription (unpublished data).

Finally, the reason underlying the lung's particular susceptibility (as opposed to other organ beds) to fluid extravasation may be as simple as extra reliance on tonic Tie-2 activation, but several other endothelial receptor/ligand pairs may also be involved. More broadly, exploring the impact of the endothelium in other pulmonary diseases may yield valuable insights [61]. Disturbances in other members of the angiopoietin axis—e.g., Ang-4, soluble Tie-2—in ARDS also need to be explored. Such studies will further define the role of the endothelium—as opposed to the epithelium—in defending the alveolar permeability barrier.

**Patient Summary**

**Background**

Sepsis is a severe illness caused by overwhelming infection of the bloodstream by toxin-producing bacteria. It results from an infection in one part of the body (such as the lungs or the skin) that has then spread throughout the body. In developed countries, the number of deaths due to sepsis has fallen in the past decades. Nevertheless, about 30% of patients admitted to hospital with sepsis still die from the disease or the secondary effects of the disease (physicians refer to these secondary effects as “complications”). One of these complications is a severe medical condition of the lungs called acute respiratory distress syndrome (ARDS). In ARDS, small blood vessels in the lungs become leaky and release fluid. This prevents the lungs from doing their job—that is, taking up oxygen and disposing of carbon dioxide. Patients experience trouble breathing and usually need to be put on respirators. If the condition lasts too long, the lung tissue becomes damaged, sometimes irreversibly. In the past few years, scientists have begun to understand what keeps blood vessels properly “sealed.” One of the potential players is a protein called Tie-2, which is present on the surface of blood vessel cells and affects their behavior. Tie-2 is itself controlled by a pair of proteins called Ang-1 and Ang-2. When the Ang-1 protein binds to Tie-2, it switches it on. When the Ang-2 protein binds to Tie-2, it switches it off.

**Why Was This Study Done?**

The researchers wanted to find out whether Tie-2, and the Ang-1 and Ang-2 proteins, might play a role in sepsis and ARDS in humans. In previous research in mice, excess levels of Ang-1 seemed to make blood vessels less leaky than they normally are, suggesting that Ang-2 might have the opposite effect. In humans, lungs have the highest Tie-2 levels of all adult organs, and reduced Tie-2 levels in the lungs have been found in some infants who died from respiratory problems.

**What Did the Researchers Do and Find?**

They tested whether excess levels of Ang-2 occur in sepsis and could cause leakiness of lung blood vessels in three different ways. First, they measured the level of Ang-2 in the blood of patients with sepsis. Second, they tested whether blood from patients with sepsis could cause leakiness in blood vessels grown in the laboratory. Finally, they tested whether elevated levels of Ang-2 affect the leakiness of blood vessels in the lungs and affect the lungs' proper functioning in mice. They found that Ang-2 levels are raised in patients with sepsis, especially in those that have impaired uptake of oxygen. During the course of the disease, Ang-2 levels mirror a patient's condition: they go up as the condition worsens and go down as the patient gets better. They then showed that treating blood vessels grown in the laboratory with blood from patients with ARDS made these blood vessels leaky. They could get the same results by treating the blood vessels with only Ang-2, and reverse the leakiness by subsequent treatment with Ang-1. Consistent with a key role of Ang-2 in ARDS, they found that injecting Ang-2 into the blood of healthy mice caused ARDS-like symptoms in the mice.

**What Does This Mean?**

These results suggest that Ang-2 might be a key player in ARDS due to sepsis. The results also suggest that measuring levels of Ang-2 in a patient might be a way for doctors to assess how much lung damage there has been in a patient who has developed ARDS. This new study raises the possibility that reducing Ang-2 levels in patients might help to prevent or improve ARDS in patients with sepsis. Additional studies in patients with sepsis, and in patients with ARDS that is not caused by sepsis, are needed to clarify the roles of Ang-2 and Tie-2 in ARDS and the suitability of Ang-2 as a target for therapy.

**Where Can I Find More Information Online?**

The following Web sites contain information on ARDS.

The ARDS Support Center:

http://www.ards.org/

MedlinePlus:

http://www.nlm.nih.gov/medlineplus/ency/article/000103.htm

ARDSNet:

http://www.ardsnet.org

Pages from the American Lung Association:

http://www.lungusa.org/site/pp.asp?c=dvLUK9O0E&b=35012

Shands Healthcare:

http://www.shands.org/health/information/article/000103.htm

## Abbreviations

Ang-1: angiopoietin-1
Ang-2: angiopoietin-2
APACHE: acute physiology and chronic health evaluation
ARDS: acute respiratory distress syndrome
EC: endothelial cell
FBS: fetal bovine serum
FiO_2_: fraction of inspired air consisting of oxygen
HMVEC: human microvascular endothelial cell
MLC: myosin light chain
MLCK: myosin light chain kinase
MLC-p: phosphorylated myosin light chain
P_a_: permeability coefficient of albumin
PaO_2_: partial pressure of oxygen in arterial blood
PVDF: polyvinylidene difluoride
VEGF: vascular endothelial growth factor
siRNA: short interfering RNA
W/D: wet-to-dry
